# Direct puncture the superior ophthalmic vein guiding by Dyna-CT to obliterating a traumatic carotid-cavernous sinus fistula: A case report and literature review

**DOI:** 10.1097/MD.0000000000031560

**Published:** 2022-10-28

**Authors:** Xue-Feng Min, Gang Yuan, Guang-Yan Si, Yan-Neng Xu

**Affiliations:** a Department of Neurosurgery, The Affiliated Traditional Chinese Medicine Hospital of Southwest Medical University, Luzhou, China; b Department of Interventional Radiology, The Affiliated Traditional Chinese Medicine Hospital of Southwest Medical University, Luzhou, China.

**Keywords:** carotid-cavernous sinus fistula, Dyna-CT, interventional therapy, superior ophthalmic vein

## Abstract

Traumatic carotid-cavernous sinus fistula (TCCF) is a pathological shunt between the carotid arteries and cavernous sinus due to trauma. Imaging-guided (e.g., ultrasonic image and fluoroscopic roadmap image) direct puncture of the superior ophthalmic vein (SOV) for embolization of TCCF has been previously described in other studies.

**Patient concerns and diagnosis:**

We report a case of TCCF in a 58-years-old male patient who was admitted to our hospital with a sustained head injury after falling from a high platform, resulting in rapidly progressive swelling, pain, diminishing vision for more than 6 months, and blindness in his left eye for 1 month.

**Interventions and outcomes:**

The patient underwent digital subtraction angiography and endovascular embolization. After the failure of super-selection of the left cavernous sinus, an alternative approach to obliterating the TCCF by puncturing the SOV is directly guided by Dyna-CT. After embolization, the patient’s clinical symptoms gradually disappeared and discharged from the hospital 5 days later. No recurrence or complications occurred during follow-up for 1 year.

**Conclusion:**

This case illustrates that direct puncture of the SOV guided by Dyna-CT as an alternative approach to embolization of TCCF is safe, effective, and feasible.

## 1. Introduction

Traumatic carotid-cavernous sinus fistula (TCCF) is a pathological shunt between the carotid arteries and the cavernous sinus by trauma.^[1]^ TCCF usually causes direct-type and high-flow arteriovenous fistulas.^[2]^ It presents not only ocular symptoms but also severe intracranial pathologies caused by ophthalmic venous hypertension. Therefore, aggressive treatment is recommended.^[3]^ Endovascular intervention became the first-line strategy for CCF treatment as the discipline of neuro-interventional radiology developed.^[4]^ One key step of TCCF treatment is the choice of the best vascular approach. Some studies showed that transvenous embolization is the safest and most effective technique for the treatment of CCF.^[1,5,6]^ However, there may be various difficult problems with the venous approach of super-selective of the cavernous sinus during interventional radiation clinically. Direct puncture of the superior ophthalmic vein (SOV) is an alternative venous approach to embolization of TCCF.^[7]^ Puncture of the SOV under imaging guidance (e.g., ultrasound, road map) is a common therapeutic strategy.^[7]^ This article presents an alternative imaging-guided method, Dyna-CT, to puncture the SOV to treat TCCF.

## 2. Case description

**Figure 1.**
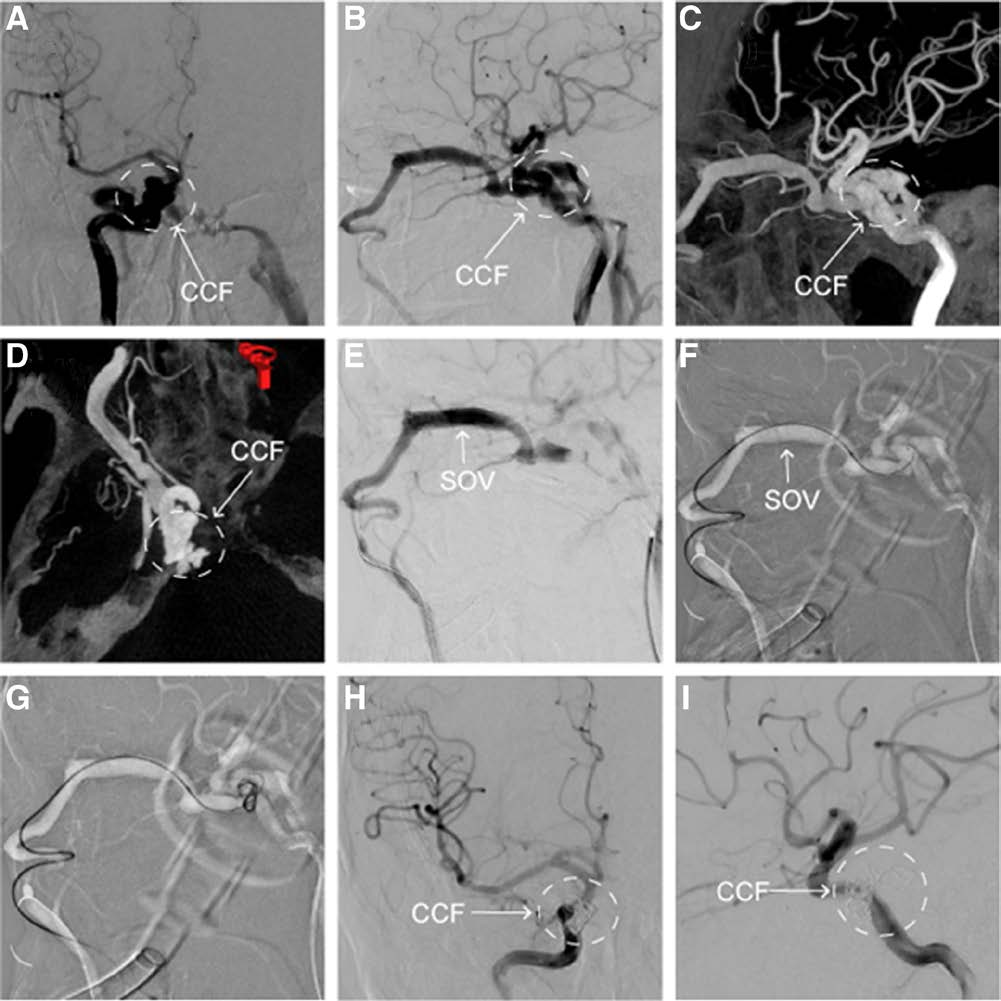
(a−d) DSA images showed the right TCCF. (e−g) DSA images showed the process of embolization *via* a transvenous approach. (h) and (i) DSA images showed the right TCCF was in complete obliteration. DSA = digital subtraction angiography, TCCF = traumatic carotid-cavernous sinus fistula.

**Figure 2.**
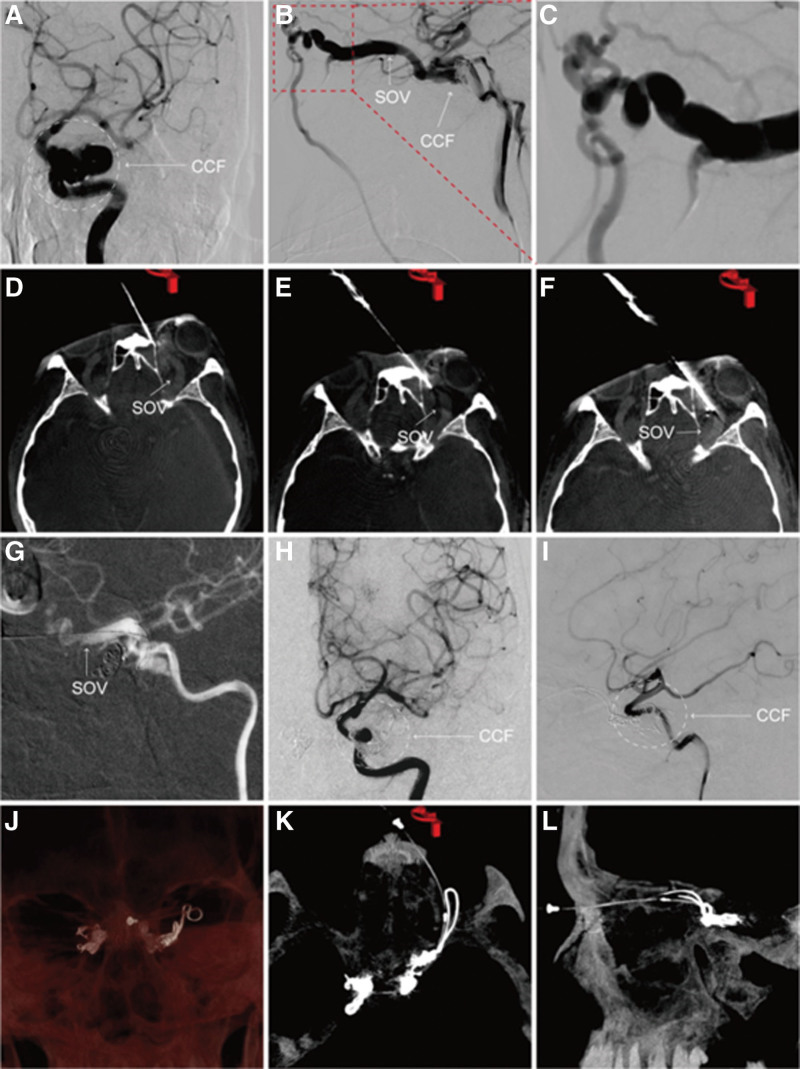
(a−b) DSA images showed that the left TCCF and the left SOV were remarkably dilated. (c) The AV was constituted with tortuous vessels. (d−f) The process of puncture the SOV guided by Dyna-CT. (g) After successful puncture the SOV, super-selective the CS and obliterated the fistula with coils. (h) and (i) Angiography images showed the fistula was completely disappearing. (j−l) Dyna-CT images were reconstructed and showed the coils in the bilateral TCCF. AV = angular vein, CS = cavernous sinuses, DSA = digital subtraction angiography, SOV = superior ophthalmic vein, TCCF = traumatic carotid-cavernous sinus fistula.

A 58-years-old man was admitted to our hospital with sustained head injury after falling from a high platform, causing rapidly progressive swelling, pain, diminishing vision for more than 6 months, and blindness in his left eye for 1 month. Physical examination showed that both eyes had complete ophthalmoplegia, ptosis, chemosis, and pulsating proptosis and were limited in all directions. The pupil size of the right eye was approximately 8 millimeter and that of the left eye was approximately 5 millimeter. Light reflection from the eyes was normal. After the patient signed the informed consent form, digital subtraction angiography confirmed bilateral direct-type TCCF. Selective angiography of the right carotid artery showed that the right CCF mainly flowed back through the ipsilateral SOV, angular vein (AV), facial vein (FV), and anterior jugular vein (AJV), with a small amount of drainage via the inter-cavernous sinuses, inferior petrosal sinus (IPS), and left internal jugular vein (IJV) (Fig. 1a−d). The left CCF mainly refluxed through the ipsilateral SOV, AV, FV, AJV, and a small amount through the IPS-left internal jugular vein (Fig. 2a−c).

Under general anesthesia, the right TCCF underwent endovascular coiling of the fistula via the right AJV, FV, SOV, and cavernous sinuses (CS) approaches, resulting in successful embolization of the fistula (Fig. 1e−i). We wanted to use the same access to embolize the left TCCF. However, the angiography image indicated that the left FV communicated with the left SOV through tortuous vessels of the AV (Fig. 2c). After repeated attempts, the 1.7 F microcatheter (Stryker, ProLwer14, SL10) and 0.014-inch micro-guidewire (Codman Neuro Scout) failed from left FV to enter the left SOV from the left AV. CT and digital subtraction angiography images suggested that the left SOV was remarkably dilated and relatively shallow. Thus, a direct puncture of the left SOV has a high probability of success. First, a scalp needle was inserted into the left inner canthus subcutaneously, approximately 0.5 centimeter from the inside to the outside, as a marker. Dyna-CT images were then reconstructed to determine the relationship between the direction of the needle tip and the target path. A puncture needle (18 G, Terumo, Japan) was used to puncture the SOV. During the puncture process, the direction and depth of the needle were adjusted according to the Dyna-CT imaging. After a successful puncture, a soft sheath was sent to the SOV (Fig. 2d−f). After super-selectivity of the left CS by the microcatheter, electric detachment coils successively filled the sinus until the fistula completely disappeared (Fig. 2g−l). The soft sheath was extracted, followed by a 10-minutes local pressing hemostasis.

After treatment, the patient’s clinical symptoms gradually disappeared, and the patient was discharged from the hospital 5 days after the endovascular intervention. No recurrence or complications occurred during the follow-up for 1 year.

## 3. Discussion

TCCF is treated with endovascular intervention, mostly using the transvenous approach (e.g., IPS or FV access). However, access is possibly absent because of thrombosis, tortuous vessels, or anatomic variations. In these cases, endovascular access to the CS through direct puncture of the dilated SOV can be a useful alternative, a technique that has been described previously by several authors.^[7,8]^

Approaches to puncture the dilated SOV include surgical exposure and image guiding.^[7]^ However, surgical exposure inevitably leads to greater trauma and requires multi-departmental team collaboration. Moreover, surgical SOV exposure may cause blepharoptosis and bilateral forehead dysesthesia.^[9]^ Compared with surgical exposure, imaging-guided percutaneous puncture of SOV is a minimally invasive method. Reviewing the literature, ultrasonic or fluoroscopic roadmaps are the main techniques used in the puncture of the SOV.^[7]^ However, orbital bone may interfere puncture during ultrasound guidance. The fluoroscopic roadmap is a 2-dimensional image with all overlapping structures. Thus, during the puncture process, the direction and depth of the needle require repeated adjustments under roadmap imaging, which increases the risk of complications. Fortunately, Dyna-CT imaging can overcome these disadvantages.

Dyna-CT, which is also known as C-arm CT, has become increasingly adopted in interventional therapy due to its high image quality, versatility, guiding, monitoring, and assessing.^[10]^ The major advantage of Dyna-CT imaging is tomographic images and has a short learning curve. Dyna-CT has been applied in different clinical disciplines for puncture guidance. Examples include guided percutaneous kidney biopsy,^[11]^ assisted percutaneous micro balloon compression for trigeminal neuralgia,^[12]^ and other complex punctures.^[13]^ However, to the best of our knowledge, Dyna-CT guided puncture of the SOV has not been reported. In our case, we used direct puncture of the left SOV to treat TCCF after the microcatheter failed through the tortuous vessels of the AV.

Although other researchers have reported complications with injury of the levator muscle and supraorbital nerve, retrobulbar hematoma, infection, granuloma, and damage to the trochlea during follow-up,^[7,14,15]^ there is no recurrence or complications during follow-up for 1 year in this case. Therefore, direct puncture of the SOV guided by Dyna-CT is just an alternative approach to obliterating TCCF only after conventional access fails. Because the patient underwent local compression and a soft sheath, there was no bleeding or hematoma in the eyelid after embolization in this case. In our experience, these principles should be followed to avoid the complications of direct puncture SOV guided by Dyna-CT. First, the SOV diameter was accurately assessed, and an appropriate puncture route was chosen. Second, the CCF should be entirely embolized to reduce recurrence and bleeding at the puncture site. Third, the soft sheath should be used routinely to reduce damage to the peripheral nerves, muscles, and other tissues.

A potential limitation of this study is that it’s only a case report and requires randomized controlled trials for further validation.

## 4. Conclusions

In conclusion, direct puncture of the SOV guided by Dyna-CT as an alternative approach to embolization of CCF is a safe, effective, and feasible method; however, further randomized controlled trials with large samples are needed to confirm this.

## Author contributions

**Conceptualization:** Yan-Neng Xu.

**Investigation:** Xue-Feng Min, Gang Yuan.

**Supervision:** Guang-Yan Si, Yan-Neng Xu.

**Writing – original draft:** Xue-Feng Min, Gang Yuan.

**Writing – review & editing:** Yan-Neng Xu.
